# Crystal Structures of a Hyperthermophilic Archaeal Homoserine Dehydrogenase Suggest a Novel Cofactor Binding Mode for Oxidoreductases

**DOI:** 10.1038/srep11674

**Published:** 2015-07-08

**Authors:** Junji Hayashi, Shota Inoue, Kwang Kim, Kazunari Yoneda, Yutaka Kawarabayasi, Toshihisa Ohshima, Haruhiko Sakuraba

**Affiliations:** 1 Department of Applied Biological Science, Faculty of Agriculture, Kagawa University, Ikenobe 2393, Miki-cho, Kagawa 761-0795, Japan; 2 Department of Biological Sciences, Graduate School of Science, Osaka University, Osaka 560-0043, Japan; 3 Department of Bioscience, School of Agriculture, Tokai University, Aso, Kumamoto, 869-1404, Japan; 4 Institute of Genetic Resources, Faculty of Agriculture, Kyushu University, Hakozaki, Fukuoka 812-8581, Japan; 5 Health Research Institute, National Institute of Advanced Industrial Science and Technology (AIST), Amagasaki 661-0974, Japan; 6 Department of Biomedical Engineering, Faculty of Engineering, Osaka Institute of Technology, 5-16-1, Ohmiya, Asahi-ku, Osaka, 535-8585, Japan

## Abstract

NAD(P)-dependent dehydrogenases differ according to their coenzyme preference: some prefer NAD, others NADP, and still others exhibit dual cofactor specificity. The structure of a newly identified archaeal homoserine dehydrogenase showed this enzyme to have a strong preference for NADP. However, NADP did not act as a cofactor with this enzyme, but as a strong inhibitor of NAD-dependent homoserine oxidation. Structural analysis and site-directed mutagenesis showed that the large number of interactions between the cofactor and the enzyme are responsible for the lack of reactivity of the enzyme towards NADP. This observation suggests this enzyme exhibits a new variation on cofactor binding to a dehydrogenase: very strong NADP binding that acts as an obstacle to NAD(P)-dependent dehydrogenase catalytic activity.

Homoserine dehydrogenase (HseDH, EC 1.1.1.3) is a key enzyme in the biosynthetic pathway from aspartate to homoserine (Hse), which is a common precursor for the synthesis of three amino acids, methionine, threonine and isoleucine in plants and microorganisms^1^,^2^,^3^. In this pathway, aspartate is first phosphorylated to β-aspartyl phosphate (β-Ap) by aspartate kinase, after which L-aspartate-β-semialdehyde (Asa) dehydrogenase catalyzes the conversion of β-Ap to Asa. The third enzyme in the pathway, HseDH, catalyzes the NAD(P)H-dependent reduction of Asa to Hse. Subsequent metabolism of Hse yields methionine, threonine or isoleucine, which are all essential in humans. Because HseDH is central to the synthesis of these amino acids, regulation of the enzyme by its end products has been extensively studied in bacteria, yeast and plants at both the activity and genetic levels^4^,^5^,^6^,^7^. In particular, threonine-dependent feedback regulation to control HseDH activity has been analyzed in detail. Additionally, HseDH is thought to be a potential target for the structure-based design of antibiotics or herbicides, as the enzyme is not present in mammals^1^,^2^,^8^. However, information about the structure of HseDH remains limited. Indeed, the crystal structures of only the *Saccharomyces cerevisiae* and *Thermus thermophilus* enzymes have so far been reported^9^,^10^.

Up to now, no HseDH from Archaea (the third domain of life) or from hyperthermophiles has been characterized. The structures of putative HseDHs from *Archaeoglobus fulgidus*, *Thermoplasma acidophilum* and *T. volcanium* were deposited in the Protein Data Bank (PDB), but the enzymological properties and structural details of these enzymes have not been reported. Within the genomic sequence of the hyperthermophilic archaeon *Pyrococcus horikoshii*, we found a gene (open reading frame identification number PH1075) whose predicted amino acid sequence exhibits 31.2% identity to that of the *A*. *fulgidus* HseDH homolog. In the present study, we expressed that gene, which encoded a HseDH homolog, characterized the enzyme produced and revealed the enzyme to be a highly thermostable HseDH. We then determined the crystal structure of this enzyme. Refinement of the structure showed the presence of a bound cofactor, NADP(H), although it was not added during crystallization, which indicates earlier high-affinity binding of the cofactor to the protein. Surprisingly, however, we found that NADP does not act as a cofactor for this enzyme, but as a strong inhibitor of NAD-dependent oxidation of Hse. We therefore used site-directed mutagenesis to analyze the cofactor-binding site of the enzyme and to evaluate the factors responsible for the NADP-mediated inhibition. Here we report the unique cofactor binding mode of *P. horikoshii* HseDH, which is not observed in conventional NAD(P)-dependent dehydrogenases.

## Results and Discussion

### Molecular and catalytic properties of *P. horikoshii* HseDH

After transforming *Escherichia coli* with pCHseDH, an expression vector encoding His-tagged HseDH, the crude extract from the recombinant cells exhibited strong HseDH activity, and the enzyme was readily purified in two simple steps: heat treatment and affinity column chromatography. About 28 mg of purified enzyme were obtained from 0.5 L of *E. coli* culture. The purified enzyme gave a single protein band on SDS-PAGE and had a specific activity (Hse oxidation) of about 114 μmol·min^–1^·mg^–1^ at 50 °C. SDS-PAGE showed the subunit molecular mass of *P. horikoshii* HseDH to be about 40 kDa, which is consistent with the molecular weight (36,925) calculated from the amino acid sequence. That the native molecular mass determined by gel filtration was about 92 kDa suggests the native enzyme is a homodimer. The enzyme was highly thermostable and retained full activity after incubation for 10 min at temperatures up to 75 °C. Even after incubation for 10 min at 95 °C, the enzyme retained 70% of its activity, making *P. horikoshii* HseDH the most thermostable HseDH described to date. Evaluation of the catalytic activity at different pH values revealed the enzyme to be maximally active at around pH 11.0. The enzyme was stable over a wide range of pH values, losing no activity when incubated at pH values between 5.0 and 12.0 for 10 min at 50 °C. *P. horikoshii* HseDH showed typical Michaelis-Menten kinetics for oxidation; the Km values for NAD and Hse were 0.32 ± 0.04 and 6.1 ± 0.1 mM, respectively.

### Overall structure and structural homologues

**Figure 1:**
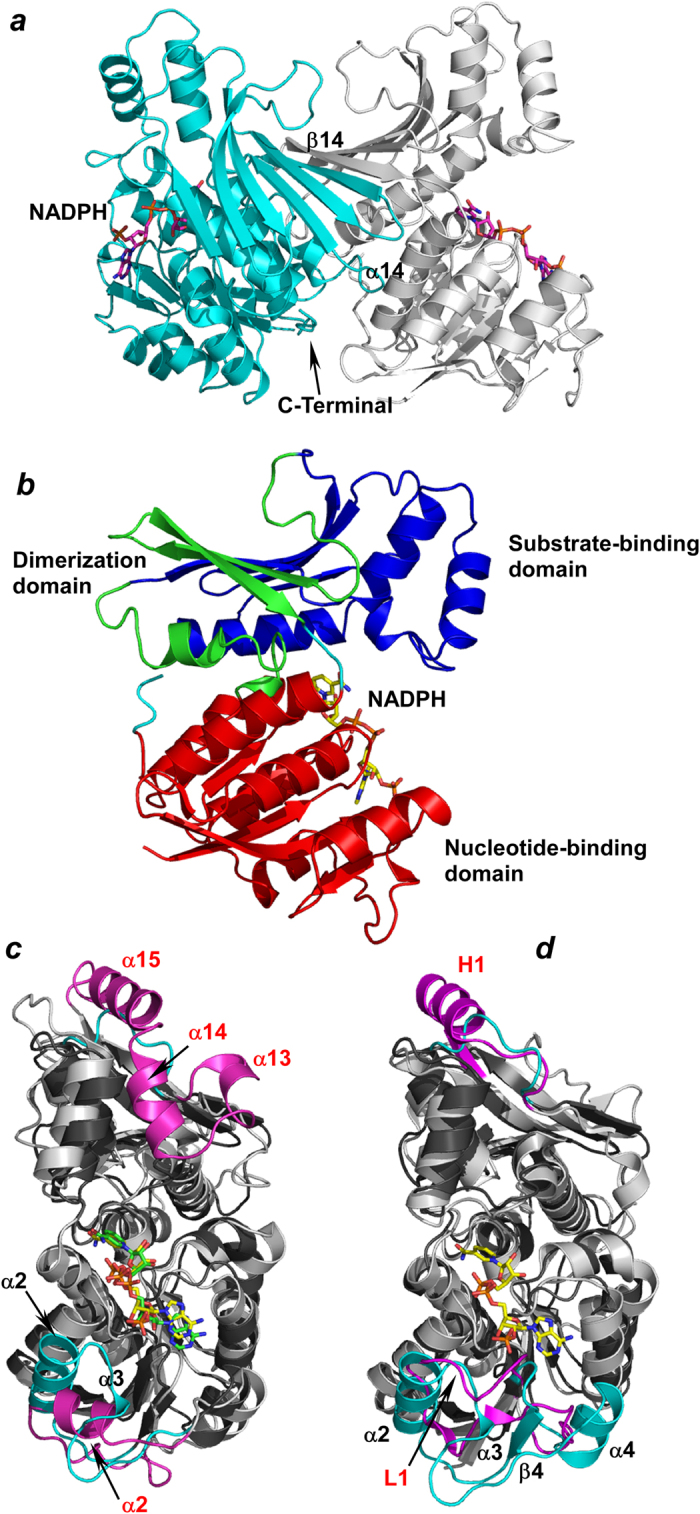
Overall structure and structural homologues. ***a***, Overall structure of *P. horikoshii* HseDH dimer. The adjacent subunit is shown in gray. The region involved in dimer formation is indicated. NADPH is shown in magenta. ***b***, Ribbon plot of the *P. horikoshii* HseDH monomer. The nucleotide-binding domain (residues 1–139 and 297–315), dimerization domain (140–160 and 266–293), and substrate-binding domain (residues 161–265) are in red, green and blue, respectively. NADPH is in yellow. ***c***, Superposition of the structures of *P. horikoshii* HseDH (dark gray) and *S. cerevisiae* HseDH (light gray). The surface elements in *P. horikoshii* HseDH (α2 and α3: cyan and black labels) and those in *S. cerevisiae* HseDH (α2, α13, α14 and α15: magenta and red labels) are shown. NADPH in *P. horikoshii* HseDH and NAD in *S. cerevisiae* HseDH are in yellow and green, respectively. ***d***, superposition of the structures of *P. horikoshii* HseDH (dark gray) and *T. thermophilus* HseDH (light gray). The surface elements in *P. horikoshii* HseDH (α2, α3, α4 and β4: cyan and black labels) and those in *S. cerevisiae* HseDH (H1and L1: magenta and red labels) are shown. NADPH in *P. horikoshii* HseDH is in yellow.

The structure of substrate-free *P. horikoshii* HseDH was determined using molecular replacement and refined at a resolution of 2.30 Å (Table 1). The asymmetric unit consisted of one homodimer with a solvent content of 70.1%, which corresponded to a Matthews coefficient^11^ of 4.1 Å^3^ Da^–1^. The model of the dimer (Fig. 1a) contained the ordered residues (1-319) in each subunit, two NADPH molecules (see below), five 2-methyl-2,4-pentanediol (MPD) molecules, two Na atoms and 212 water molecules. The two nearly identical subunits (root-mean-square deviation (r.m.s.d.) = 0.35 Å) were closely associated mainly through hydrophobic interactions around α14, β14 and the C-terminal region (residues 314–318) in both subunits. The surface area buried in the dimer interface was 1600 Å^2^ (calculated using a probe with a radius of 1.4 Å with the “Protein interfaces, surfaces and assemblies” service at the European Bioinformatics Institute (http://www.ebi.ac.uk/pdbe/prot_int/pistart.html))^12^, which is 11% of the total surface area of the enzyme. Each monomer consisted of three domains: a nucleotide-binding domain (residues 1–139 and 297–315), a dimerization domain (140–160 and 266–293) and a substrate-binding domain (residues 161–265) (Fig. 1b). When the model of the *P. horikoshii* HseDH monomer was sent to the DALI server^13^ for identification of proteins with similar structures (as of December 2, 2014), the three proteins with the highest structural similarity were putative HseDH homologs from *A. fulgidus* (PDB entry 3DO5, r.m.s.d. 1.9 Å; Joint Center for Structural Genomics, unpublished work), *T. volcanium* (PDB entries 3C8M and 3JSA, r.m.s.d.s between 1.9 and 2.0 Å; Midwest Center for Structural Genomics, unpublished work) and *T. acidophilum* (PDB entry 3ING, r.m.s.d. 2.1 Å ; Joint Center for Structural Genomics, unpublished work), which was expected.

**Figure 2:**
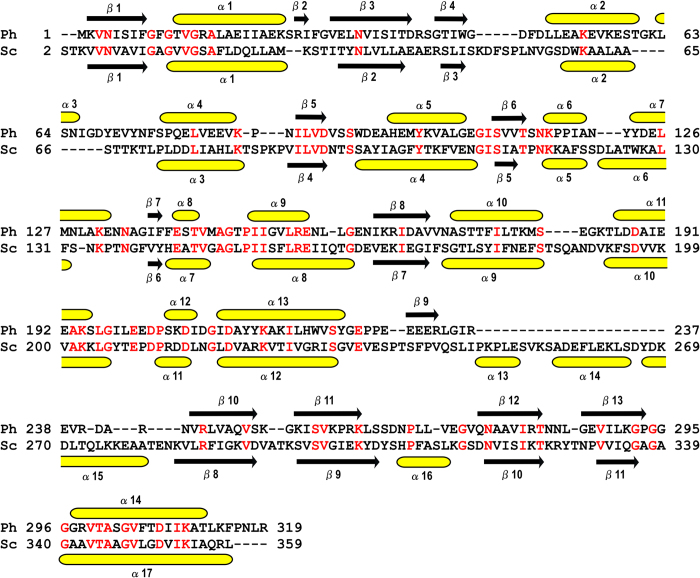
Structure-based amino acid sequence alignment of *P. horikoshii* HseDH (Ph) and *S. cerevisiae* HseDH (Sc). The conserved residues are shown in red. The secondary structural assignments for *P. horikoshii* HseDH are shown above the alignment; those for *S. cerevisiae* HseDH are shown below the alignment.

The *P. horikoshii* HseDH monomer also showed high structural similarity to *S. cerevisiae* HseDH (PDB entries 1EBU and 1EBF, r.m.s.d.s between 2.1 and 2.2 Å^10^) (Fig. 1c), as did the assembled dimer. In addition, the Na ion-binding site of *S. cerevisiae* HseDH is well conserved in *P. horikoshii* HseDH. In the *S. cerevisiae* enzyme, the coordination sphere surrounding the ion consists of the backbone carbonyl atoms of Glu143, Val146, Ala148 and Leu150, and a side-chain oxygen of Glu143. Of these residues, Glu143, Val146 and Ala148 are conserved as Glu140, Val143 and Ala145, respectively, in *P. horikoshii* HseDH, but Leu150 is replaced by Thr147. On the other hand, we found that three surface α-helices (α13, α14 and α15), which are located at the top of the substrate-binding domain of *S. cerevisiae* HseDH, are absent in *P. horikoshii* HseDH (Fig. 1c). The sequence alignment also showed that 22 residues in the corresponding region in *S. cerevisiae* HseDH are not present in *P. horikoshii* HseDH (Fig. 2). Another noteworthy difference was in the structure of the chain located at the bottom of the nucleotide-binding domain. In *S. cerevisiae* HseDH, the chain (residues 47–68) contains only one short α-helix (α2) and is positioned far away from the cofactor NAD. By contrast, in *P. horikoshii* HseDH the corresponding region (residues 46–72) contains helices α2 and α3, and the C-terminal part of α2 extends toward the cofactor-binding site, providing an important component (Lys57) for NADP(H) binding (see below).

The crystal structure of *T. thermophilus* HseDH has reportedly been solved in both the substrate/cofactor-free form (1.4 Å resolution) and Hse-bound binary form (2.0 Å resolution)^9^. Unfortunately, however, coordinate files for these structures are not available (as of December 2, 2014), though a 1.7 Å-resolution structure (PDB entry 2EJW) of the substrate/cofactor-free enzyme has been deposited in PDB. When we compared the structure of the *P. horikoshii* HseDH monomer with that of *T. thermophilus* HseDH (2EJW-A), we found that the main chain coordinates of the former were basically the same as those of the latter (r.m.s.d. 2.5 Å) (Fig. 1d). At the top of the substrate-binding domain, however, one helix (H1) that corresponds to α15 of *S. cerevisiae* HseDH is present in the *T. thermophilus* HseDH structure. Furthermore, the structure of the chain located at the bottom of the nucleotide-binding domain is entirely different in the two enzymes. The large surface loop containing β4, α2, α3 and α4 in *P. horikoshii* HseDH is replaced by a small loop in *T. thermophilus* HseDH, though part of the loop (L1; residues 44–50) extends toward the cofactor-binding site, as in the corresponding part in *P. horikoshii* HseDH.

### Cofactor binding site

Our initial electron density map for the substrate-free *P. horikoshii* HseDH showed an extra density within the nucleotide-binding site and, after construction and refinement of the peptide chain, an NADP(H) molecule could be modeled into that density. No cofactor was present during crystal growth, but this molecule may have originated in the *E. coli* cells and was then retained throughout the protein purification. To identify the bound molecule, the mass spectra of the cofactor released from wild-type *P. horikoshii* HseDH were measured in a low *m/z* range using Fourier transform ion cyclotron mass spectrometer (FT-ICR MS) as described in the Methods section. In addition, as controls, both NADP and NADPH were employed as potential binding compounds with *P. horikoshii* HseDH, and their exact masses were measured using the same FT-ICR MS. When 1-μM samples of NADP and NADPH were introduced into FT-ICR MS, their single charged *m/z* values were detected as 742.067 and 744.083, respectively, in the negative mode (see Supplementary Fig. S1 online). The mass of the cofactor released from wild-type HseDH was 744.082 *m/z*, which is consistent with the mass of NADPH. On the other hand, the mass of NADP was not detected in *P. horikoshii* HseDH. These results demonstrate that the molecule bound to *P. horikoshii* HseDH is NADPH.

**Figure 3:**
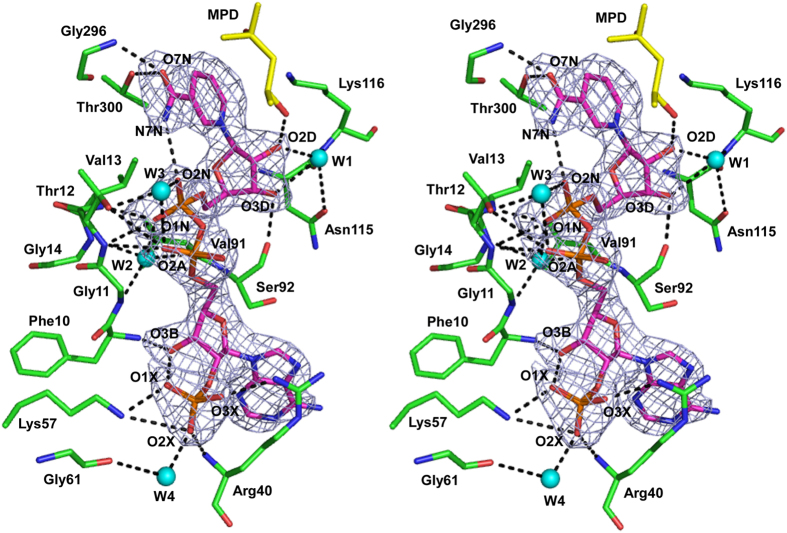
Stereographic close-up of NADPH bound to *P. horikoshii* HseDH. The networks of hydrogen bonds are shown as dashed lines. Residues that interact with NADPH are shown in green. NADPH is in magenta, and MPD is in yellow. W1-W4 indicate water molecules. The final σ_A_-weighted Fo - Fc omit electron density map for NADPH is shown at the 3σ level. Oxygen, phosphate and nitrogen atoms are shown in red, orange and blue, respectively.

**Figure 4:**
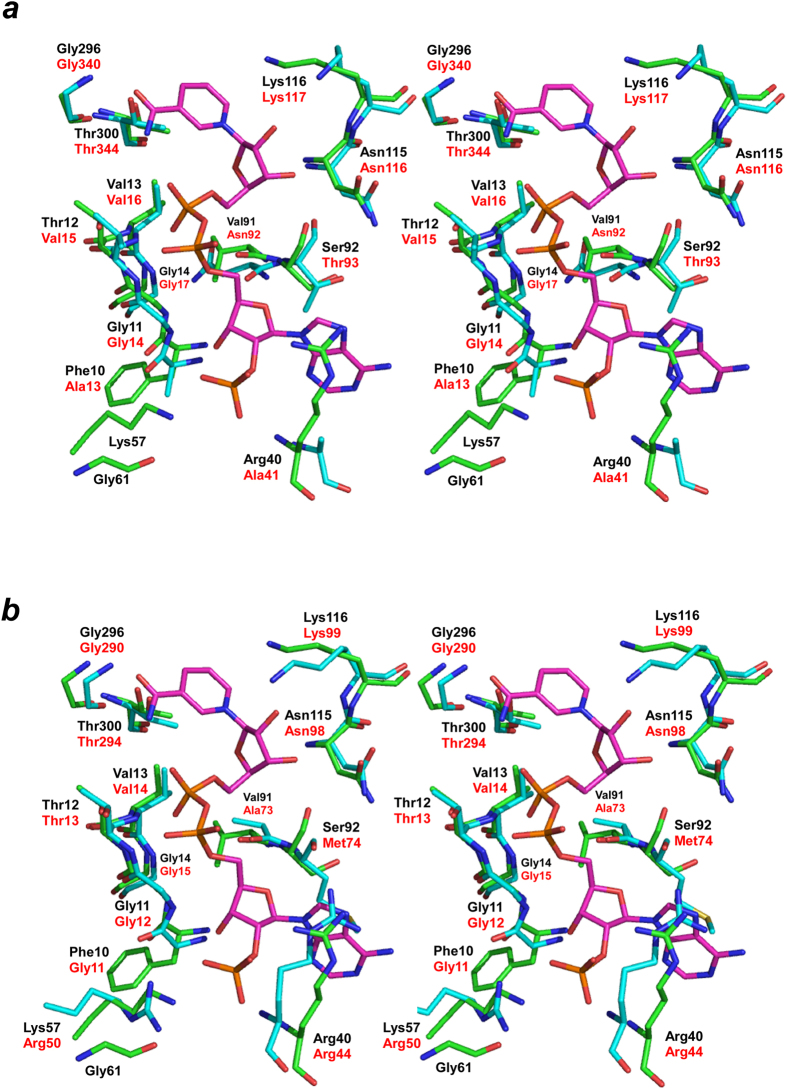
Cofactor binding site. ***a***, Comparison of the nucleotide-binding site structures in *P. horikoshii* HseDH (green and black labels) and *S. cerevisiae* HseDH (cyan and red labels) (stereo representation). ***b***, Comparison of the nucleotide-binding site structures in *P. horikoshii* HseDH (green and black labels) and *T. thermophilus* HseDH (cyan and red labels) (stereo representation). NADPH in *P. horikoshii* HseDH is shown in magenta. Atoms are colored as in Fig. 3.

The electron density corresponding to the NADPH bound within the nucleotide-binding site of *P. horikoshii* HseDH was very clear, enabling us to place the ligand with reasonable accuracy (Fig. 3). Three hydrogen-bonding interactions are made between the nicotinamide amide group (O7N and N7N) and the backbone nitrogen of Gly296, the side-chain of Thr300 and the nicotinamide phosphate (O2N). Via a water molecule (W1), the C2 and C3 hydroxyl groups (O2D and O3D) of the nicotinamide ribose interact with the backbone nitrogen of Lys116 and the side chain of Asn115. The O2D and O3D also interact with O4 of the MPD molecule and the backbone O atom of Ser92, respectively. The nicotinamide phosphate (O1N) interacts with the backbone N atoms of Thr12 and Val13, and via a water molecule (W2), the O1N also interacts with the backbone O atom of Val91 and the backbone N atoms of Gly11 and Gly14. Also via a water molecule (W3), the O2N of the nicotinamide phosphate interacts with the side chain of Thr12. The O2A of the adenine phosphate forms a hydrogen bond with W3, in addition to interactions with the backbone N atom and side chain of Thr12. The C3 hydroxyl group (O3B) of the adenine ribose interacts with the backbone N atom of Phe10 and the C2 phosphate group (O1X) of the adenine ribose. No hydrogen bonding interactions are made between the adenine base and the enzyme. By contrast, the C2 phosphate group (O1X, O2X, and O3X) of the adenine ribose is tightly held at its position through interactions with the side chain and backbone N atom of Arg40, the side chain of Lys57, and via a water molecule (W4), the backbone O atom of Gly61. Most of the interactions between the enzyme and the NADH moiety of the cofactor are conserved in the NAD-bound *S. cerevisiae* HseDH structure (1EBF-A), although Thr12, Phe10, Val91 and Ser92 in *P. horikoshii* HseDH are replaced by Val15, Ala13, Asn92 and Thr93, respectively, in *S. cerevisiae* HseDH (Fig. 4a). By contrast, the residues interacting with the C2 phosphate group of the adenine ribose in *P. horikoshii* HseDH are not conserved in *S. cerevisiae* HseDH. Arg40 in the former is replaced with Ala41 in the latter. In addition, Lys57 and Gly61 in *P. horikoshii* HseDH are absent from *S. cerevisiae* HseDH, because both residues belong to the C-terminal part of the helix α2, which specifically extends toward the cofactor-binding site in *P. horikoshii* HseDH. On the other hand, comparison of the cofactor-binding sites between *P. horikoshii* HseDH and substrate/cofactor-free *T. thermophilus* HseDH indicates that the residues interacting with the cofactor in the *P. horikoshii* enzyme are likely more strictly conserved in the *T. thermophilus* enzyme, though Phe10, Val91 and Ser92 in *P. horikoshii* HseDH are respectively replaced by Gly11, Ala73 and Met74 in *T. thermophilus* HseDH (Fig. 4b). In contrast to the situation in *S. cerevisiae* HseDH, the two residues (Arg44 and Arg50) that respectively correspond to Arg40 and Lys57 in *P. horikoshii* HseDH are observed in *T. thermophilus* HseDH. These residues belong to L1, which extends toward the cofactor-binding site in *T. thermophilus* HseDH, and their side chains are thought to be situated at positions where they can interact with the C2 phosphate group of the adenine ribose. That said, superposition of *T. thermophilus* HseDH onto *P. horikoshii* HseDH showed that Arg44 of *T. thermophilus* HseDH would sterically hinder the binding of NADP(H) (Fig. 4b), which means structural changes would occur upon NADP(H) binding to the *T. thermophilus* HseDH. Thus, the cofactor-bound structure of *T. thermophilus* HseDH will be necessary for further analysis of the cofactor-enzyme interactions of this enzyme.

### Insight into the inhibition by NADP

As mentioned, NADPH was observed in the crystal structure of *P. horikoshii* HseDH, though it was not added during crystallization. Because this suggests earlier high-affinity binding of the cofactor to the protein, we assessed the electron-acceptor specificity for Hse oxidation catalyzed by *P. horikoshii* HseDH. To our surprise, the enzyme did not utilize NADP as a cofactor at all under the standard assay conditions. In addition, native-PAGE of the enzyme followed by activity staining confirmed that *P. horikoshii* HseDH has no reactivity toward NADP (see Supplementary Fig. S2 online). We next tested NADP’s ability to inhibit NAD-dependent Hse oxidation. The double-reciprocal plots of *v* versus the NAD concentration at several fixed concentrations of NADP suggest competitive inhibition, but the Ki value for NADP is extremely low. When curve-fitting using Morrison’s equation^14^, which is often employed for tight-binding inhibitors, was carried out using Prism (GraphPad Software, La Jolla, CA, USA), the Ki value for NADP was calculated to be 5.2 ± 0.1 nM (see Supplementary Fig. S3 online). These results indicate that NADP does not act as the cofactor for *P. horikoshii* HseDH, but as the strong inhibitor of NAD-dependent Hse oxidation.

Within *P. horikoshii* HseDH, the C2 phosphate group of the cofactor adenine ribose is tightly held at the nucleotide-binding site through three direct hydrogen bonding interactions with the side chains of Arg40 and Lys57. To reduce the number of these interactions, we constructed R40A and K57A mutants, which had striking effects on the enzymes reactivity against NADP (Table 2). In particular, K57A substitution had a strongly positive effect on NADP kinetics, with the enzyme exhibiting a Vmax^NADP^ value (43.3 ± 0.3 μmol·min^–1^·mg^–1^) less than half the Vmax^NAD^ of the wild-type enzyme (114 ± 7.6 μmol·min^–1^·mg^–1^). The mutant enzyme retained a Vmax^NAD^ of 78.6 ± 0.3 μmol·min^–1^·mg^–1^, which is about 70% of the wild-type Vmax^NAD^. The Km value for NADP was estimated to be 0.06 ± 0.01 mM, which is almost one-fifth that for NAD exhibited by the wild-type enzyme (0.32 ± 0.04 mM). These results indicate that the K57A mutant retains high affinity for NADP. The reason why the Km value for NAD is also reduced to 0.05 ± 0.01 mM in K57A is unclear. Taken together, these results suggest that the inhibition by NADP is caused by its strong binding to *P. horikoshii* HseDH.

### Hse/NADPH-bound structure

When FT-ICR MS analysis was performed with both the R40A and K57A mutants, the mass of NADPH was also detected in both proteins, whereas the mass of NADP was not detected (see Supplementary Fig. S4 online). The NADPH peak intensity of R40A mutant was equal to that of wild type HseDH, indicating that NADPH tightly bound to R40A, as in the case of the wild type enzyme. By contrast, a weak NADPH peak intensity was detected in K57A. This suggests that the interaction between NADPH and enzyme is weaker in the K57A mutant, compared with those in the wild-type and R40A enzymes. As mentioned below, the cofactor was also observed in the structure of the K57A mutant. Prior to mass analysis, buffer in the enzyme solution was replaced with 10 mM ammonium acetate using PD-10 desalting column (see Methods). Therefore, most of NADPH molecules bound to the K57A mutant may be removed from the protein by this pre-treatment.

**Figure 5:**
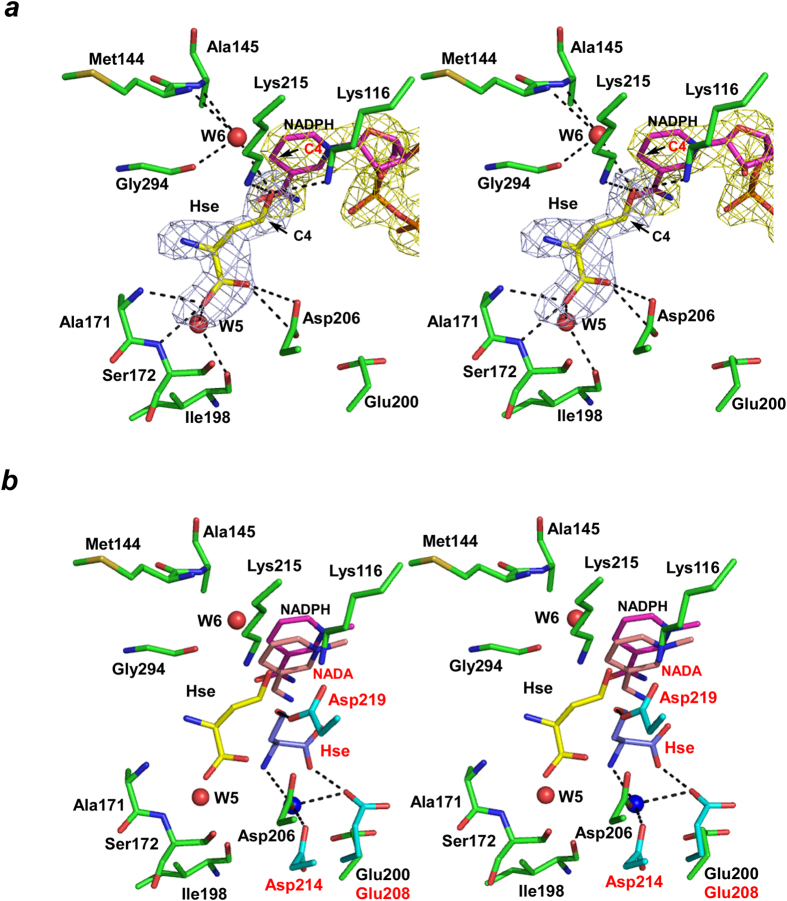
Substrate binding site. ***a***, Stereographic close-up of the Hse-binding site in *P. horikoshii* HseDH. The networks of hydrogen bonds are shown as dashed lines. W5 and W6 indicate water molecules. NADPH and Hse are in magenta and yellow, respectively. The C4 atoms of the pyridine ring (red) and Hse (black) are labeled. The final σ_A_-weighted Fo - Fc omit electron density maps for Hse (light blue) and NADPH (yellow) are shown at the 3σ level. ***b***, Comparison of active site structures in *P. horikoshii* HseDH (green and black labels) and *S. cerevisiae* HseDH (cyan and red labels) (stereo representation). NADPH, Hse and waters in *P. horikoshii* HseDH are shown in magenta, yellow and red, respectively. NADA, Hse and water in *S. cerevisiae* HseDH are in pink, light blue and blue, respectively. The networks of hydrogen bonds in *S. cerevisiae* HseDH are shown as dashed lines. Oxygen and nitrogen atoms are in red and blue, respectively.

When K57A mutant was cocrystallized with NADPH, diffraction quality crystals could not be obtained. In the presence of Hse without NADPH, however, a few diffraction quality crystals were obtained. In addition, cocrystallization of K57A with NADPH (1 mM) and Hse (1 mM) has been carried out, but the diffraction quality crystals could not be reproduced. Addition of excess NADPH may prevent the formation of a stable K57A/NADPH/Hse complex. The model of the Hse-bound K57A mutant was refined at a resolution of 2.43 Å (Table 1). The asymmetric unit consisted of one homodimer with a solvent content of 46.1%, which corresponded to a Matthews coefficient^11^ of 2.3 Å^3^ Da^–1^. The model of the dimer contained the ordered residues (1–319) in each subunit, two NADPH molecules, two Hse molecules, two Na atoms and 211 water molecules. As with the wild-type enzyme, we observed a clear density corresponding to the cofactor bound within the nucleotide-binding site. Because the cofactor was not present during crystal growth, this molecule might have originated in the *E. coli* cells. As described above, NADPH, but not NADP, was detected in K57A upon FT-ICR MS analysis. We therefore placed an NADPH molecule at this position. Moreover, we also observed an extra density within the active site cavity, and after construction and refinement of the peptide chain, a Hse molecule could be modeled into that density (Fig. 5a).

Within our model, the carboxyl group of Hse hydrogen bonded with the side-chain of Asp206 and the backbone N atoms of Ala171 and Ser172 and, via a water molecule (W5), with the backbone O atom of Ile198. The O atom of the C4 hydroxyl group interacts with the side-chains of Lys116 and Lys215 and, via another water molecule (W6), with the backbone N atoms of Met144 and Ala145 and the backbone O atom of Gly294. The C4 atom of Hse is situated in front of the C4 atom of the nicotinamide ring, which enables hydride transfer between the two.

The structure of *S. cerevisiae* HseDH in complex with Hse and 3-aminopyridine adenine dinucleotide (NADA), an NAD analogue, has been reported. When the Hse/NADA-bound *S. cerevisiae* HseDH structure (1EBU-D) was superimposed on the Hse/NADPH-bound K57A structure, we found that the position of the bound substrate and the amino acid residues involved in substrate binding totally differed between the two enzymes (Fig. 5b). Notably, the side-chain of Glu208 in *S. cerevisiae* HseDH forms a hydrogen bond with the carboxyl group of Hse, whereas the positioning of the corresponding residue (Glu200) in our model is too far away to interact with the substrate. Within the structure of Hse-bound *T. thermophilus* HseDH, the orientation of the bound substrate also reportedly differs from that in *S. cerevisiae* HseDH, and Glu180, which corresponds to Glu208 in the *S. cerevisiae* enzyme (Glu200 in *P. horikoshii* HseDH), forms a hydrogen bond with the amino group of Hse^9^. This indicates that the orientation of the bound Hse substantially differs among the three enzyme structures. The structures of Hse/NADPH-bound K57A and Hse/NADA-bound *S. cerevisiae* HseDH have been solved at resolutions of 2.4 Å and 2.6 Å, respectively, and the Hse-binding mode in *T. thermophilus* HseDH was described on the basis of an Hse/enzyme binary complex (2.0 Å-resolution structure). Therefore, a high-resolution structure of the Hse/cofactor/enzyme ternary complex may be required to precisely determine the binding mode of Hse in HseDH.

**Figure 6:**
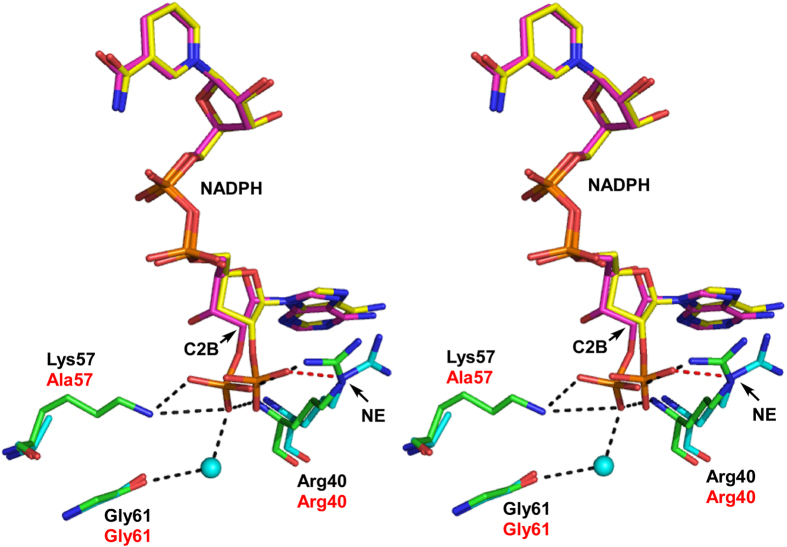
Comparison of the NADPH-binding site structures in *P. horikoshii* HseDH wild-type (green and black labels) and the K57A mutant (cyan and red labels) (stereo representation). NADPH molecules in wild-type and K57A are shown in magenta and yellow, respectively. The hydrogen bonds around the C2 phosphate group of the adenine ribose are shown as black dashed lines in the wild-type protein and a red dashed line in the K57A mutant. The C2B atom in NADPH and HE atom in Arg40 are labeled. Atoms are colored as in Fig. 3.

Superposition of the Hse/NADPH-bound K57A structure onto the NADPH-bound wild-type structure showed that the NADPH molecule in the mutant structure was positioned/configured nearly identically to the NADPH molecule in the wild-type structure, except for the positioning of the C2 phosphate group of the adenine ribose (Fig. 6). As described above, the C2 phosphate is tightly held in position through five surrounding hydrogen bonds in the wild-type enzyme. In K57A mutant, however, the C2 phosphate group is rotated in a clockwise direction around C2B of NADPH by about 30° relative to the wild-type structure. In addition, the guanidino group of Arg40 in the mutant is also rotated clockwise by about 90° around the NE atom of Arg40 relative to the wild-type structure. As a result, only one interaction between O3X of the C2 phosphate and the NE of Arg40 was observed at the corresponding position in K57A (Fig. 6). Nearly all the interactions between the cofactor NADH moiety and the enzyme are conserved between the two enzymes, except for a few water-mediated interactions. Given that no substantive difference in the domain organization of the two enzymes was found, these observations suggest that the larger number of interactions around the C2 phosphate of the cofactor strengthen the affinity of wild type HseDH for NADP, making the binding too tight to utilize NADP as the cofactor.

### Insight into cofactor preference

At present, it is unclear why *P. horikoshii* HseDH structure includes NADPH not NADP. We performed kinetic analysis for the *in vivo* forward reaction of HseDH (Asa/NADH and Asa/NADPH). Interestingly, both NADH and NADPH were able to serve as the coenzyme for *P. horikoshii* HseDH, although the reaction rate with NADPH (Vmax^NADPH^ = 8.7 ± 1.5 μmol·min^–1^·mg^–1^) was only 3.0% of that with NADH (Vmax^NADH^ = 295 ± 66 μmol·min^–1^·mg^–1^). The Km values for NADH and NADPH were estimated to be 0.019 ± 0.01 mM and 0.015 ± 0.01 mM, respectively, indicating that NADH is favored as the natural cofactor. These results suggest that the enzyme activity is regulated by changes in the NADH/NAD and NADPH/NADP ratios. However, further detailed investigation is required to prove this hypothesis.

Because NAD differs from NADP only in the C2 phosphate group of the adenine ribose, the amino acid residues interacting with this region are thought to be responsible for the cofactor specificity of the enzymes^15^,^16^. In the medium-chain dehydrogenase/reductase family enzymes, the coenzyme binding domains possess similar β-α-β motifs centered around a highly conserved GXGXXG/A sequence. Among the amino acid residues of this β-α-β motif, the primary determinant of NAD specificity is the presence of an Asp or Glu residue, which forms hydrogen bonds with both the C2 and C3 hydroxyl groups of the NAD adenine ribose and occupies the space that would be occupied by the C2 phosphate group of the NADP adenine ribose. In NADP dependent enzymes, on the other hand, this residue is usually replaced by a smaller residue such as Gly, Ala or Ser, accompanied by one or more positively charged residues, Arg and/or Lys, which form a binding pocket for the C2 phosphate group. Similar features are also observed in short-chain dehydrogenase/reductase family enzymes^17^. In some enzymes with dual cofactor specificity, replacement of the hydrogen bonds associated with each cofactor binding has been observed^18^,^19^,^20^. HseDH belongs to an expansive and diverse class of oxidoreductases, and although the overall fold of the catalytic region is unique^10^, the nucleotide-binding domain conforms to the Rossmann fold^21^,^22^, like conventional NAD(P)-dependent dehydrogenases. The structure of the nucleotide-binding site in our model indicates that *P. horikoshii* HseDH would have a strong preference for NADP. However, NADP does not act as the cofactor for this enzyme, but as a strong inhibitor for NAD-dependent Hse oxidation. To the best of our knowledge, this is the first reported example of an enzyme in which the very strong binding of NADP is an obstacle to its catalytic activity, in this case NAD(P)-dependent dehydrogenase activity. Although the physiological significance of the inhibitory effect of NADP remains unclear, the present study indicates that the molecular details underlying cofactor preference in NAD(P)-dependent dehydrogenases are more complex than expected, and the cofactor specificity cannot be predicted even from the structural information in the absence of biochemical data.

## Methods

### **P**rotein expression and site-directed mutagenesis

The gene encoding HseDH (PH1075, the gene information is available in the Kyoto Encyclopedia of Genes and Genomes database) was amplified by PCR. The oligonucleotide primers used to amplify the HseDH gene fragment were 5′-ACCTTTC**CATATG**AAAGTTAACATCTC-3′, which contains a unique *Nde*I restriction site (bold) overlapping the 5′ initiation codon, and 5′-TTA**AAGCTT**CTAGCGGAGGTTTGGGAAT-3′, which contains a unique *Hin*dIII restriction site (bold) proximal to the 3′ end of the termination codon. Chromosomal *P. horikoshii* DNA was isolated as described previously^23^ and used as the template. The amplified 1.0-kb fragment was digested with *Nde*I and *Hin*dIII and ligated with the expression vector pColdI (TAKARA BIO, Japan) previously linearized with *Nde*I and *Hin*dIII to generate pCHseDH, which was then used to transform *E. coli* strain BL21 (DE3) codon plus-RIPL (Agilent Technologies, Santa Clara, CA, USA). The transformants were cultivated at 37 °C in 0.5 L of Luria-Bertani medium until the optical density at 600 nm reached 0.5, after which expression was induced by addition of 1 mM isopropyl-β-D-thiogalactopyranoside to the medium, and cultivation was continued for an additional 24 h at 15 °C. Site-directed mutagenesis was accomplished using a QuikChange Lightning site-directed mutagenesis kit (Agilent Technologies) according to the manufacturer’s instructions. pCHseDH served as the template, and the following set of oligonucleotides was used as the mutagenic primers (the mutations are underlined): 5′-CATCTCAATAACCGACGCAAGCGGGACTATATGGGG-3′and 5′-CCCCATATAGTCCCGCTTGCGTCGGTTATTGAGATG-3′ for R40A, and 5′-GGAAGCTAAGGAAGTTGCGGAATCCACGGGAAAGC -3′ and 5′-GCTTTCCCGTGGATTCCGCAACTTCCTTAGCTTCC -3′ for K57A.

### Purification and enzymatic characterization

Cells were harvested by centrifugation, suspended in 10 mM Tris-HCl buffer (pH 7.5) (buffer A) and disrupted by sonication, after which the cell debris was removed by centrifugation (15,000 × *g* for 10 min). The resulting supernatant, which served as the crude extract, was heated at 90 °C for 10 min, and the denatured proteins were removed by centrifugation (15,000 × *g* for 10 min). The supernatant from that step was loaded onto a nickel-charged Chelating Sepharose Fast Flow column (GE Healthcare Bio-Sciences AB, Uppsala, Sweden) that had been equilibrated with buffer A. The column was then washed with the same buffer, and the enzyme was eluted with a linear gradient of 0–0.5 M imidazole in the buffer. The active fractions were collected and dialyzed against buffer A and used for biochemical experiments. Expression and purification of the R40A and K57A mutants were performed using the same method described for His-tagged wild-type HseDH. To prepare the enzyme for crystallization, the active fractions eluted from the Chelating Sepharose column were pooled, concentrated, loaded onto a Superdex 200 26/60 column (GE Healthcare) previously equilibrated with buffer A and eluted with the same buffer. The active fractions were then pooled and concentrated by ultrafiltration (Amicon Ultra 30 K NMWL, MILLIPORE) for crystallization trials. Enzyme activity was assayed spectrophotometrically using a Shimadzu UV-mini 1240 spectrophotometer equipped with a thermostat. The standard reaction mixture for Hse oxidation consisted of 100 mM K_2_HPO_4_-K_3_PO_4_ buffer (pH 11.0) containing 50 mM Hse, 5 mM NAD and the enzyme in a final volume of 1.0 ml. For Asa reduction (*in vivo* forward reaction), the reaction mixture consisted of 100 mM K_2_HPO_4_-KH_2_PO_4_ buffer (pH 7.5) containing 2 mM Asa (GlysoSyn/Callaghan Innovation, Lower Hutt, New Zealand), 0.15 mM NADH (or NADPH) and the enzyme in a final volume of 1.0 ml. After warming the reaction mixture by incubation for 3 min at 50 °C without the enzyme, the reaction was started by addition of the enzyme. The appearance and disappearance of NAD(P)H was monitored from the absorbance at 340 nm (extinction coefficient ε = 6.22 mM^−1^·cm^−1^). The protein concentration was determined using the Bradford method, with bovine serum albumin serving as the standard^24^. Native-PAGE was carried out at room temperature on 7.5% polyacrylamide gel using the method of Davis^25^. Activity staining was performed at 60 °C using a mixture containing 200 mM Tris-HCl buffer (pH 7.5), 100 mM Hse, 0.04 mM phenazine methosulfate, 0.1 mM *p*-iodonitrotetrazolium violet and 10 mM NAD or NADP. SDS-PAGE (12.5% acrylamide slab gel, 1-mm thick) was carried out using the procedure of Leammli^26^, after which the protein band was stained with Coomassie brilliant blue R-250. The molecular mass of the purified enzyme was determined using a Superose 6 10/300 GL column (GE healthcare) with 10 mM Tris-HCl (pH 7.5) containing 0.2 M NaCl as the elution buffer. Gel filtration standards (Bio-Rad Lab., Hercules, CA, USA) were used as the molecular mass standards. The subunit molecular mass was determined by SDS-PAGE using eight marker proteins (6–175 kDa) (New England Biolabs Inc.). To determine the effect of temperature on its stability, the enzyme was incubated for 10 min at different temperatures in 10 mM Tris-HCl buffer (pH 7.5). The residual activity was then determined using the standard assay method. To determine the effect of pH on its stability, the enzyme was incubated in buffers at various pHs for 10 min at 50 °C, and the remaining activity was again determined using the standard assay method. The buffers (250 mM) used for these assays were acetate (pH 4.2–5.0), potassium phosphate (pH 5.7–7.5), glycylglycine (pH 8.0–9.2), and K_2_HPO_4_-K_3_PO_4_ (pH 10.1–11.6). The same K_2_HPO_4_-K_3_PO_4_ buffer was also used to determine the optimal pH for enzyme activity at 50 °C. To determine the kinetic parameters, the initial velocity was examined by varying the concentration of one substrate while keeping the concentrations of the other substrates constant, as previously described^27^.

### Structure determination

Crystals of substrate-free HseDH were obtained using the sitting-drop vapor diffusion method, in which a 1-μl drop of 12.0 mg/ml protein solution was mixed with an equal volume of mother liquor composed of 0.1 M potassium phosphate buffer (pH 6.2) and 20% MPD. The crystals were grown for 2 days at 20 °C. Single-wavelength (1.0 Å) data were collected using an ADSC CCD detector system on the NE3A beamline at the Photon Factory, Tsukuba, Japan. The measurements were carried out on a crystal cooled to 100 K in a stream of nitrogen gas. Cryoprotection was performed with 30% (v/v) MPD. Crystals of Hse-bound K57A mutant enzyme were grown in sitting drops composed of 2 μl of enzyme solution (10 mg/ml) containing 1 mM Hse mixed with 2 μl of mother liquor containing 16% polyethylene glycol monomethyl ether 2000 and 0.1 M citrate buffer (pH 6.5). The crystals were grown for 7 days at 20 °C. Diffraction data were collected at room temperature because of the large mosaicity under cryo-conditions. The crystal was mounted in a thin-walled glass capillary tube and data (1.5418 Å) were collected on an R-AXIS VII imaging-plate detector using a rotating copper-anode in-house generator (MicroMax007, Rigaku, Japan) operating at 40 kV and 20 mA. In all cases, the data were processed using HKL2000^28^. The structure of the substrate-free HseDH was solved to a resolution of 2.30 Å by molecular replacement using the MOLREP program^29^ in the CCP4 program suite^30^; the structure of *A. fulgidus* HseDH homologue (PDB entry 3DO5) served as the search model. In the final refined model, R = 0.168 (Rfree = 0.204). The structure of Hse-bound K57A mutant was solved to a resolution of 2.43 Å by molecular replacement using MOLREP^29^ with the substrate-free enzyme structure as the search model. In the final model, R = 0.164 (Rfree = 0.223). In both cases, model building was performed using the program Coot^31^, and refinement was carried out using REFMAC5^32^ and CNS^33^. Water molecules were incorporated using Coot^31^. Model geometry was analyzed using RAMPAGE^34^, and the fractions (%) of residues in the favored/allowed/outliers regions of the Ramachandran diagram were 97.5/2.5/0 and 97.8/2.2/0 for substrate-free HseDH and Hse-bound K57A mutant, respectively. The data collection and refinement statistics are listed in Table 1. Molecular graphics figures were created using PyMOL (http://www.pymol.org/).

### Mass analysis using a fourier transform ion cyclotron mass spectrometer

Prior to mass analysis, 10 mM Tris-HCl buffer (pH 7.5) in enzyme solution was replaced with 10 mM ammonium acetate using PD-10 desalting column (GE Healthcare). A sample (1 μM) of purified wild type HseDH, R40A or K57A was dissolved in 50% acetonitrile and 50 mM ammonia, after which the solution was kept at room temperature for 10 min to separate the cofactor from the protein. A solariX FT-ICR MS shielded with 9.4 Tesla superconducting magnets (Bruker Daltonics Inc., MA, USA) was employed to analyze the mass spectra of the cofactors with high mass measurement accuracy. The sample solutions were introduced into the mass spectrometer using electrospray ionization (ESI), and the mass spectra were measured in the negative mode at a 120 μl/h syringe flow rate, 200 °C drying gas temperature, 3200 V capillary voltage, and –100 voltage spray shield. For data acquisition by the solariX FT-ICR MS, free induction decay was set to 1 M size, and masses were detected in the range of 60 to 1500 *m/z*. One hundred mass spectra were combined to obtain an average spectrum for each sample. As controls, NADP and NADPH solutions in 10 mM ammonium acetate were prepared. Samples (1 μM) of NADP and NADPH were then dissolved in 50% acetonitrile and 50 mM ammonia, and their masses were analyzed using the same methods as the case of the protein samples.

## Additional Information

**Accession codes:** The atomic coordinates and structure factors (codes 4XB1 and 4XB2) have been deposited in the Protein Data Bank (http://www.rcsb.org).

**How to cite this article**: Hayashi, J. *et al.* Crystal Structures of a Hyperthermophilic Archaeal Homoserine Dehydrogenase Suggest a Novel Cofactor Binding Mode for Oxidoreductases. *Sci. Rep.* **5**, 11674; doi: 10.1038/srep11674 (2015).

## Supplementary Material

Supplementary Information

## Figures and Tables

**Table 1:** Statistics on data-collection and refinement. Values in parentheses: highest resolution data shell, r.m.s.d.: root-mean-square deviation.

	NADPH-bound wild type (PDB entry 4XB1)	Hse/NADPH-bound K57A (PDB entry 4XB2)
**Data collection**		
Wavelength (Å)	1.0	1.5418
Space group	*P*4_1_	*P*2_1_
		
**Unit cell parameters**		
	a = b = 112.6 Å	a = 78.4 Å
	c = 95.9 Å	b = 47.8 Å
		c = 90.5 Å
		β = 95.5°
Resolution range (Å)	50–2.30 (2.38–2.30)	50–2.43 (2.52–2.43)
Total No. of reflections	402797	101949
No. of unique reflections	53097	25243
Multiplicity	7.6 (7.5)	4.0 (3.4)
Completeness (%)	99.8 (99.9)	99.0 (95.7)
*R*_merge_^a^	0.051 (0.285)	0.045 (0.175)
< *I*/*σ*(*I*) >	21.4 (9.1)	15.0 (4.2)
		
**Refinement**		
Resolution range (Å)	50–2.30	50–2.43
*R*/*R*_free_ (%)^b^	16.8/20.4 (23.1/29.3)	16.4/22.3 (21.8/29.7)
No. of protein atoms	4916	4908
No. of water molecules	212	211
No. of ligands	Na^+^ions, 2	Na^+^ions, 2
	NADPH, 2	NADPH, 2
	MPD, 5	Hse, 2
		
**B-factor (Å**^b^)		
Protein	46.7	37.3
Na^+^ion	20.6	19.1
Water	43.5	33.5
NADPH	44.4	36.8
MPD	58.5	—
Hse	—	30.0
		
**R. m. s. d.**		
Bond lengths (Å)	0.020	0.014
Bond angles (°)	2.1	1.7

^a^*R*_merge_ = ∑_*hkl*_ ∑_*i*_ | *I*_*i*_(*hkl*) – ⟨I(*hkl*)⟩ | / ∑_*hkl*_ ∑_*i*_ *Ii*(*hkl*), where I_*i*_(hkl) is the scaled intensity of the *i*th observation of reflection *hkl*. ⟨I(*hkl*)⟩ is the mean value and summation over all measurements.

^b^*R*_free_ calculated with randomly selected reflections (5%).

**Table 2:** Kinetic parameters of wild type, R40A, and K57A for NAD and NADP. Assays were performed as described under “Methods”.

Enzyme	NAD				NADP			
	V_max_	K_m_	k_cat_	k_cat_/K_m_	V_max_	K_m_	k_cat_	k_cat_/K_m_
	*μmol/min/mg*	*mM*	*s*^*−1*^	*s*^*−1*^*·mM*^*−1*^	*μmol/min/mg*	*mM*	*s*^*−1*^	*s*^*−1*^*·mM*^*−1*^
Wild type	114 ± 7.6	0.32 ± 0.04	70.1 ± 4.5	219 ± 13	0	—	0	—
R40A	156 ± 6.5	0.95 ± 0.15	96.1 ± 4.6	102 ± 12	5.5 ± 0.2	0.04 ± 0.01	3.4 ± 0.2	89.1 ± 0.9
K57A	78.6 ± 0.3	0.05 ± 0.01	48.5 ± 0.2	1020 ± 60	43.3 ± 0.3	0.06 ± 0.01	26.7 ± 0.2	518 ± 29
