# Supplementary material for: Crystal Structures of a Hyperthermophilic Archaeal Homoserine Dehydrogenase Suggest a Novel Cofactor Binding Mode for Oxidoreductases
Source: Sci Rep. 2015 Jul 8;5:11674. doi: 10.1038/srep11674 (PMC4495429; doi:10.1038/srep11674)
Supplement: Supplementary Information [file srep11674-s1.pdf]

Supplementary Information

Crystal Structures of a Hyperthermophilic Archaeal Homoserine Dehydrogenase Suggest a Novel Cofactor Binding Mode for Oxidoreductases

Junji Hayashi[1], Shota Inoue[1], Kwang Kim[2], Kazunari Yoneda[3], Yutaka Kawarabayashi[4], Toshihisa Ohshima[5] and Haruhiko Sakuraba[1*]

[1] Department of Applied Biological Science, Faculty of Agriculture, Kagawa University, Ikenobe 2393, Miki-cho, Kagawa 761-0795, Japan

[2]Department of Biological Sciences, Graduate School of Science, Osaka University, Osaka 560-0043, Japan,

[3]Department of Bioscience, School of Agriculture, Tokai University, Aso, Kumamoto, 869-1404, Japan

[4]Institute of Genetic Resources, Faculty of Agriculture, Kyushu University, Hakozaki, Fukuoka 812-8581, Japan.

[5]Department of Biomedical Engineering, Faculty of Engineering, Osaka Institute of Technology, 5-16-1, Ohmiya, Asahi-ku, Osaka, 535-8585, Japan

[*]**Corresponding author**: Haruhiko Sakuraba

Department of Applied Biological Science, Faculty of Agriculture, Kagawa University, Ikenobe 2393, Miki-cho, Kagawa 761-0795, Japan , Tel.: +81-87-891-3078; Fax: +81-87-891-3078;

E-mail: sakuraba@ag.kagawa-u.ac.jp

Fig. S1: Mass spectra for NADP, NADPH and the cofactor bound to HseDH.

Fig. S2: Activity staining of purified *P. horikoshii* HseDH.

Fig. S3: Inhibition analysis.

Fig. S4: Mass spectra for the cofactor bound to wild type HseDH, R40A and K57A.

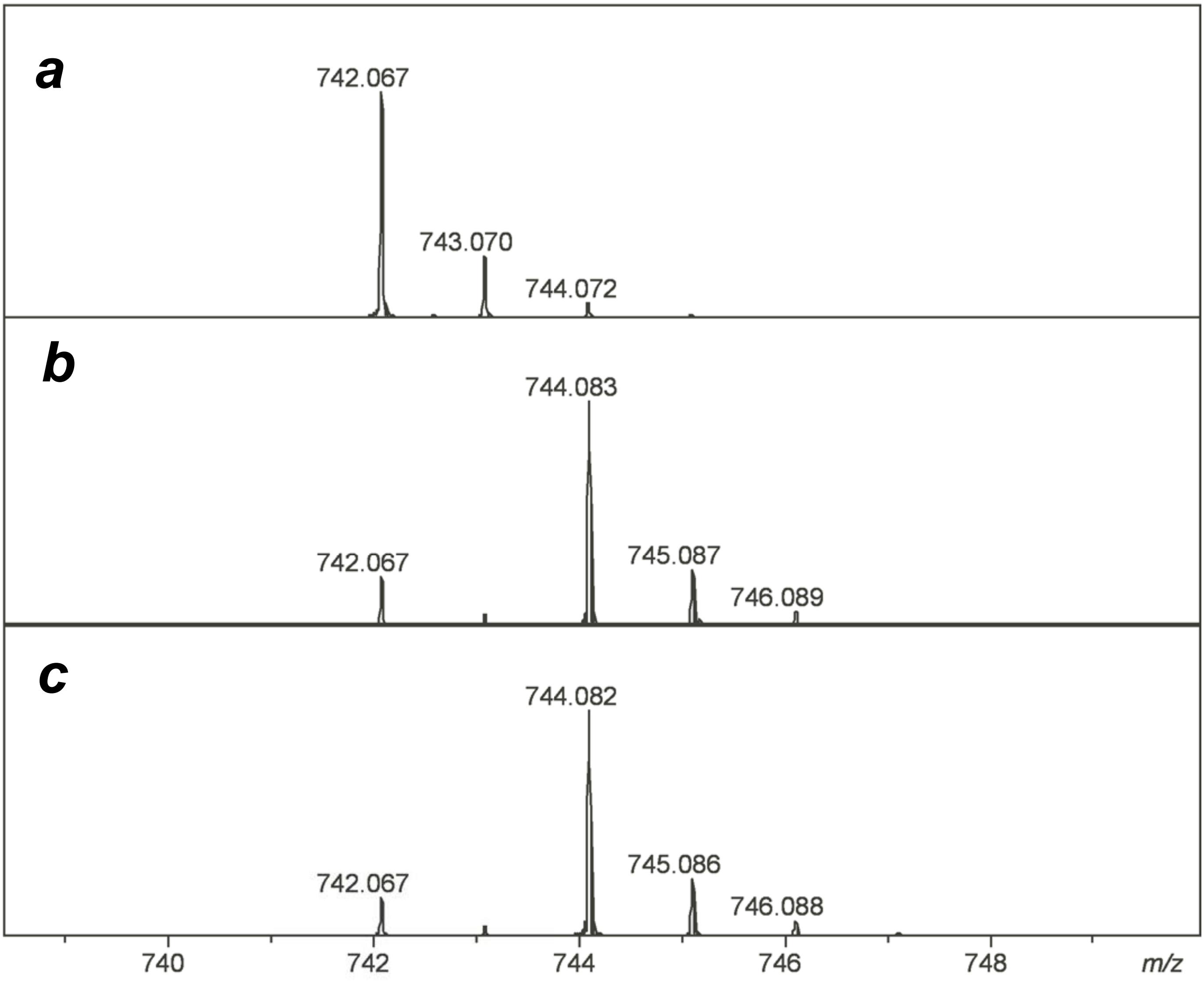

Fig. S1 Mass spectra for NADP, NADPH and the cofactor bound to HseDH.

Each mass spectrum was generated using ESI FT-ICR (9.4 T) mass spectrometry.
a, Mass spectrum for 1 μM NADP (control) ($C_{21}H_{29}N_7O_{17}P_3$, [M-2H] =742.067078 m/z).
b, Mass spectrum for 1 μM NADPH (control) ($C_{21}H_{30}N_7O_{17}P_3$, [M-H] =744.082728 m/z).
c, Mass spectrum for the cofactor bound to 1 μM purified wild type HseDH.

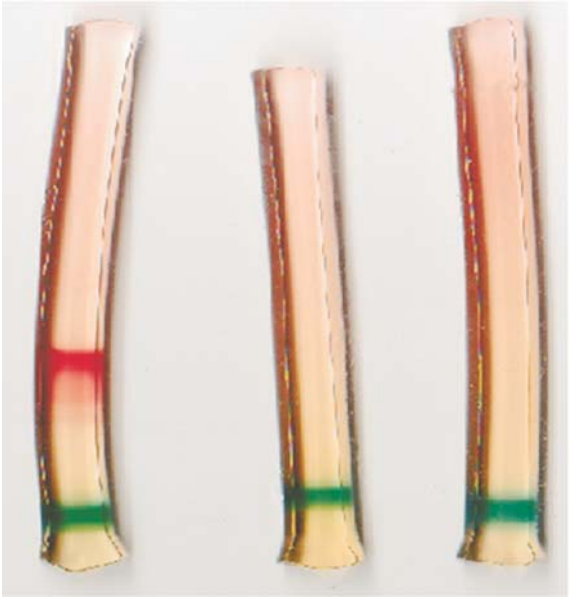

NAD NADP No cofactor

Fig. S2 Activity staining of purified *P. horikoshii* HseDH*.*

Native-PAGE was carried out at room temperature on a 7.5% polyacrylamide gel. Activity staining was performed at 60°C using a mixture containing 200 mM Tris-HCl buffer (pH 7.5), 100 mM Hse, 0.04 mM phenazine methosulfate, 0.1 mM p-iodonitrotetrazolium violet and 10 mM NAD or NADP.

a

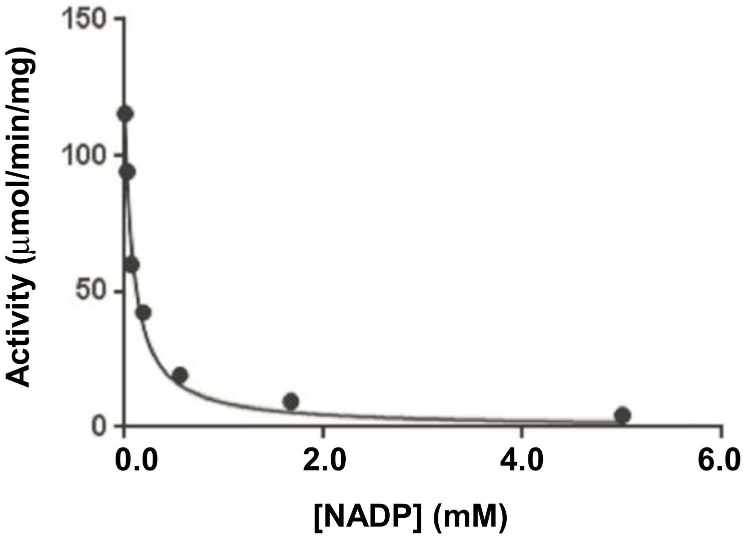

b

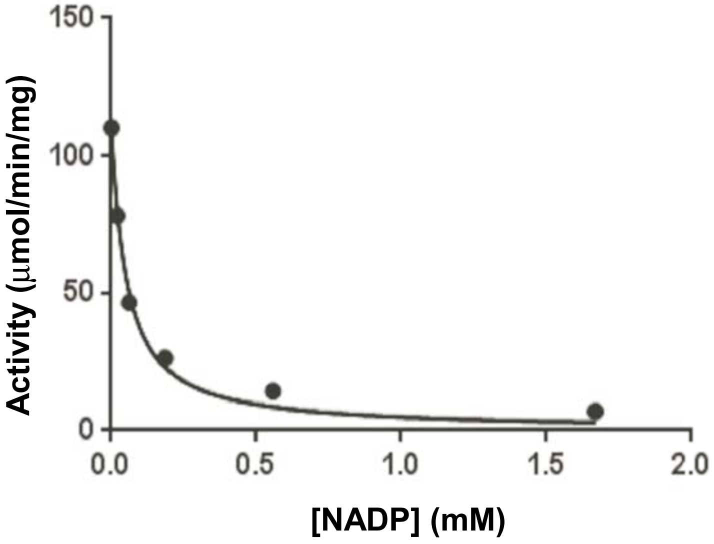

Fig. S3 Inhibition analysis*.*

***a*, Cuve-fitting of activity vs. NADP concentrations at 5 mM NAD.**
***b*, Cuve-fitting of activity vs. NADP concentrations at 2.5 mM NAD.**
Et (concentration of enzyme catalytic sites) = 0.0135 μM, Km for NAD = 320 μM
The Ki value was determined to be 5.2 ± 0.1 nM based on Morrison's equation [14] using Prism (GraphPad Software, La Jolla, CA, USA).

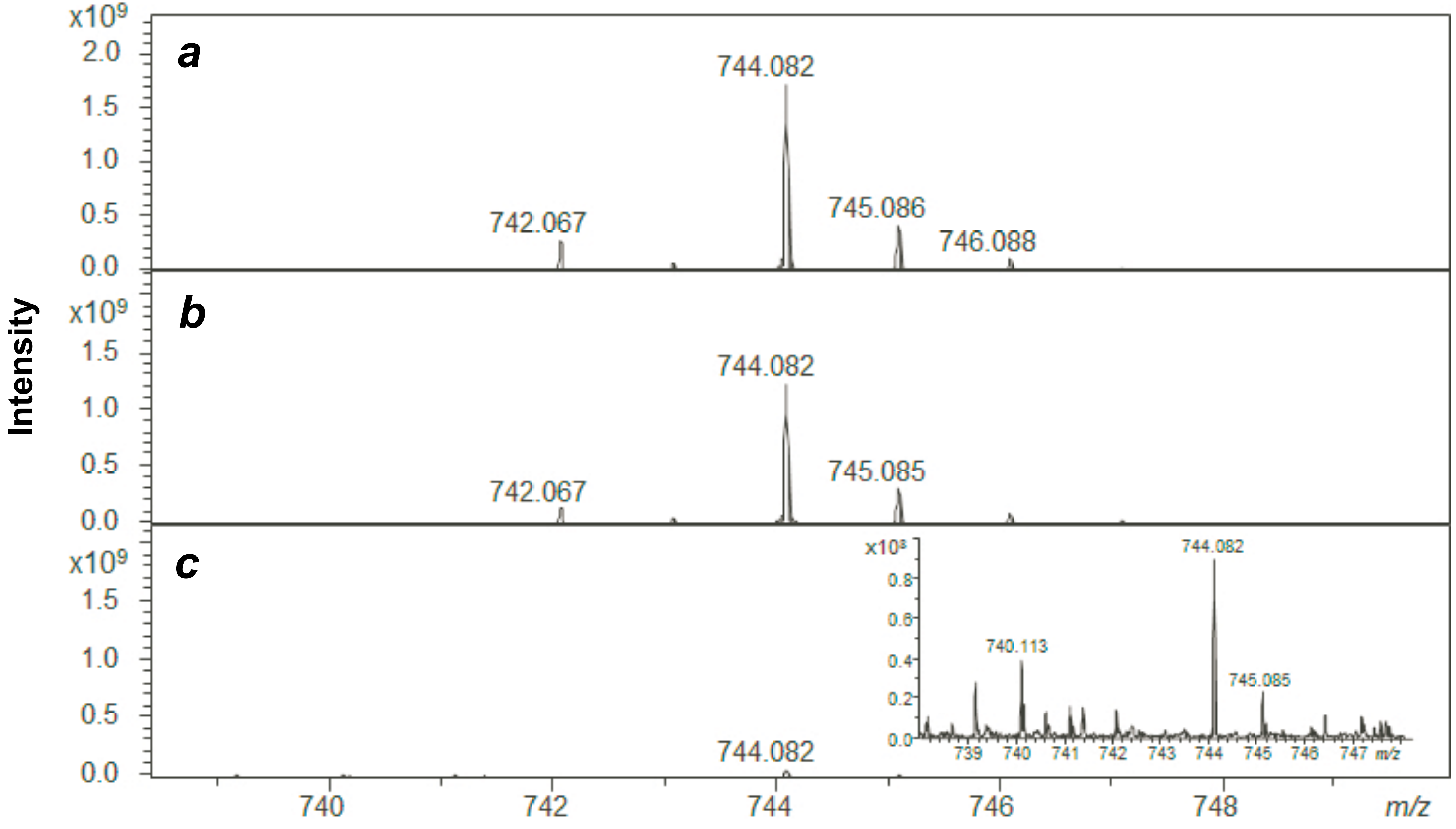

Fig. S4 Mass spectra for the cofactor bound to wild type HseDH, R40A and K57A.

Each mass spectrum was generated using ESI FT-ICR (9.4 T) mass spectrometry.
a, Mass spectrum for the cofactor bound to 1 μM purified wild type HseDH.
b, Mass spectrum for the cofactor bound to 1 μM purified R40A.
c, Mass spectrum for the cofactor bound to 1 μM purified K57A.
Inlet spectrum showed detail peak intensities of target m/z range, 738 to 748 m/z.
